# Do associations of sex, age and education with transport and leisure-time physical activity differ across 17 cities in 12 countries?

**DOI:** 10.1186/s12966-019-0894-2

**Published:** 2019-12-03

**Authors:** Josef Mitáš, Ester Cerin, Rodrigo Siqueira Reis, Terry L. Conway, Kelli L. Cain, Marc A. Adams, Grant Schofield, Olga L. Sarmiento, Lars B. Christiansen, Rachel Davey, Deborah Salvo, Rosario Orzanco-Garralda, Duncan Macfarlane, Adriano Akira F. Hino, Ilse De Bourdeaudhuij, Neville Owen, Delfien Van Dyck, James F. Sallis

**Affiliations:** 1 Institute of Active Lifestyle, Faculty of Physical Culture, Palacký University, Olomouc, Czech Republic; 2 Mary Mackillop Institute for Health Research, Australian Catholic University, Melbourne, Victoria Australia; 3 School of Public Health, The University of Hong Kong, Pok Fu Lam, Hong Kong, China; 4 Prevention Research Center, Brown School, Washington University in St. Louis, Saint Louis, MO USA; 5 Urban Management Graduate Program, Pontifical Catholic University of Parana, Curitiba, Brazil; 6 Department of Family Medicine and Public Health, University of California San Diego, La Jolla, CA USA; 7 Global Institute of Sustainability, College of Health Solutions, Arizona State University, Phoenix, AZ USA; 8 Faculty of Health and Environmental Sciences, AUT University, Auckland, New Zealand; 9 School of Medicine, Universidad de los Andes, Bogotá, Colombia; 10 Department of Sports Science and Clinical Biomechanics, University of Southern Denmark, Odense, Denmark; 11 Health Research Unit, University of Canberra, Canberra, Australia; 12 University Public of Navarra, Pamplona, Spain; 13 Centre for Sports and Exercise, University of Hong Kong, Pok Fu Lam, Hong Kong; 14 Health Technology Graduate Program, Pontifical Catholic University of Parana, Curitiba, Brazil; 15 Department of Movement and Sport Sciences, Faculty of Medicine and Health Sciences, Ghent University, Gent, Belgium; 16 Baker Heart and Diabetes Institute, Melbourne, Australia; 17 Swinburne University of Technology, Melbourne, Australia; 18 Research Foundation Flanders, Brussels, Belgium

**Keywords:** Social epidemiology, International health, Physical activity domain, IPAQ, Health disparities

## Abstract

**Background:**

Leisure-time and transport activity domains are studied most often because they are considered more amenable to intervention, but to date evidence on these domains is limited. The aim of the present study was to examine patterns of socio-demographic correlates of adults’ leisure-time and transport physical activity and how these associations varied across 17 cities in 12 countries.

**Methods:**

Participants (*N* = 13,745) aged 18–66 years in the IPEN Adult study and with complete data on socio-demographic and self-reported physical activity characteristics were included. Participants reported frequency and duration of leisure-time and transport activities in the last 7 days using the self-administered International Physical Activity Questionnaire-Long Form. Six physical activity outcomes were examined in relation with age, education, and sex, and analyses explored variations by city and curvilinear associations.

**Results:**

Sex had the most consistent results, with five of six physical activity outcomes showing females were less active than males. Age had the most complex associations with self-report transport and leisure-time physical activity. Compared to older people, younger adults were less likely to engage in transport physical activity, but among those who did, younger people were likely to engage in more active minutes. Curvilinear associations were found between age and all three leisure-time physical activity outcomes, with the youngest and the oldest being more active. Positive associations with education were found for leisure-time physical activity only. There were significant interactions of city with sex and education for multiple physical activity outcomes.

**Conclusions:**

Although socio-demographic correlates of physical activity are widely studied, the present results provide new information. City-specific findings suggest there will be value in conducting more detailed case studies. The curvilinear associations of age with leisure-time physical activity as well as significant interactions of leisure-time activity with sex and education should be further investigated. The findings of lower leisure-time physical activity among females as well as people with low education suggest that greater and continued efforts in physical activity policies and programs tailored to these high-risk groups are needed internationally.

## Background

Increasing physical activity (PA) has been identified by the United Nations [1] and World Health Organization [2] as one of four key strategies for reducing global epidemics of non-communicable diseases (NCDs). NCDs and low levels of PA are common among countries of low, medium, and high incomes [3–9]. Worldwide, physical inactivity is more prevalent among females and linearly increases with ageing populations [10]. Understanding the correlates and determinants of PA provides evidence that can be used to guide the development of interventions and policies targeted to benefit higher-risk groups [11]. Relatively consistent correlates have been documented at individual (biological, psychological), social, and built environmental levels of influence [11]. Among the most widely studied correlates are socio-demographic variables of sex, age, and education [11, 12], and these variables can be used to identify subgroups at highest risk for inactivity.

Correlates often differ by the domain of PA; i.e., leisure-time, transport, occupation, and household [12, 13]. Leisure-time and transport activity domains are studied most often because they are considered more amenable to intervention. Psychological and built environment correlates and determinants differ substantially by these two domains [11], but less attention has been paid to differences in demographic correlates between these domains of PA. There has been little research examining variations in these associations across countries. Prior studies examined demographic correlates of overall PA [14], walking [15], and sitting [16], but to date evidence on domain-specific leisure-time and transport activity is limited to national studies or comparisons between populations from high-income countries.

The International Physical Activity and Environment Network (IPEN) Adult study was the first opportunity to examine relationships between demographic correlates and domain-specific activities of adults across diverse countries. IPEN was a multi-country cross-sectional epidemiologic study using a common design and comparable methods [17]. The data from both middle- and high-income countries allowed enhanced analyses of the domain-specific correlates of physical activity internationally. The aim of the present study was to examine patterns of socio-demographic correlates of adults’ leisure-time and transport physical activity and how these associations varied across diverse countries.

## Methods

### Study design

IPEN participants were recruited from 17 cities across 12 countries: Australia (Adelaide, AU), Belgium (Ghent, BE), Brazil (Curitiba, BR), Colombia (Bogota, CO), Czech Republic (Olomouc and Hradec Králové, CZ), Denmark (Aarhus, DK), Hong Kong/China (HK), Mexico (Cuernavaca, MX), New Zealand (North Shore, Waitakere, Wellington, and Christchurch, NZ), Spain (Pamplona, ES), the United Kingdom (Stoke-on-Trent, UK), and the United States of America (Seattle/King County, Washington and Baltimore, Maryland region, US). The IPEN Adult study was designed to maximize variability in neighborhood environmental attributes related to walking and socioeconomic status (SES) by recruiting participants from neighborhoods stratified a priori for high/low walkability and high/low SES [17].

Using objective geographical information systems (GIS) data, a walkability index [18] was used to stratify neighborhood areas, except in Spain where neighborhoods were stratified based on their construction date (a proxy measure of walkability). The smallest administrative or census unit that represented a neighborhood-level geographic sector was selected for the development of the walkability measures. Administrative units were ranked into deciles based on the normalized walkability index and on neighborhood-level SES data drawn from the census (e.g., household income, education attainment, or an index) in each city. The walkability index and census-based SES scores were crossed to produce four neighborhood quadrants: high walkable / high SES; high walkable / low SES; low walkable / high SES; and low walkable / low SES. The details for each country can be found elsewhere [17].

### Participant recruitment

Households in the selected neighborhoods were identified using databases from commercial and government sources in most cities. In each selected household, an adult was invited to complete a survey and wear an accelerometer, with study dates ranging from 2002 to 2011. More information on participant recruitment can be found elsewhere [17]. Each country obtained ethical approval from their local institutional review boards, and all participants provided informed consent.

### Participants

The entire IPEN Adult study consisted of 14,222 adults aged 18–66 years. The current study examined data from 13,745 participants from 17 study cities across 12 countries with complete data on socio-demographic and self-reported PA characteristics.

### Measures

#### Socio-demographic characteristics

Self-reported socio-demographic variables included age, sex, education, working status, and marital status. Years of education were categorized into ‘less than high school’, ‘high school graduate’ and ‘college degree or more’. Working status was recoded as working or not, and marital status was dichotomized into living as a couple versus not.

#### Physical activity

This study employed the self-administered International Physical Activity Questionnaire-Long Form (IPAQ-LF), which was a validated measure designed for international use [14, 19]. The IPAQ-LF collected data on reported frequency and duration (bouts of at least 10 min) of moderate and vigorous activities for each domain (occupation, transport, household, and leisure-time) over the last seven days. For present analyses, only summary scores for leisure-time and transport activity were used because not all countries collected data on occupation and household PA. Three outcomes for each domain were calculated to reflect some of the complexity of PA patterns. First, engaging ≥10 min/week in the PA domain with a binary outcome (no vs. yes) identified people who reported any PA in the domain. Second, duration of PA in the domain (only including respondents with non-zero minutes of PA) provided a continuous measure of PA in each domain. Third, engaging in ≥150 min/week of PA in the domain was a binary outcome that identified participants who engaged in a substantial amount of PA, sufficient to achieve the recommended weekly amount in a single domain of PA.

### Data analytic plan

Descriptive statistics (relative frequencies, means, standard deviations and percentages of missing values) were computed for all variables for the whole sample and by city. Associations of age, educational attainment and sex with PA outcomes, and the moderating effects of city, were estimated using generalized additive mixed models (GAMMs) [20]; accounting for clustering effects at the administrative unit level [21]. GAMMs with binomial variance and logit link functions were used for the binary outcomes (e.g., not engaging vs. engaging in ≥150 min/week of leisure-time PA), while GAMMs with Gamma variance and logarithmic link functions were used for the two continuous PA outcomes (to account for the fact that the outcome could only assume positive values and residuals were positively skewed). Each measure of weekly minutes of PA was operationalized as a binary (engagement in ≥10 min/week of PA) and a continuous variable (non-zero min/week of PA) because preliminary analyses indicated that the number of zero values was significantly greater than that expected under a Gamma distribution. The reported exponential function of the regression coefficients of the GAMMs for binary outcomes represent odds ratios, while those of the GAMMs for continuous outcomes represent the proportional increase in non-zero weekly minutes of PA associated with 1 unit increase in the predictor.

Main-effect GAMMs estimated the relationships of age, sex, and educational attainment with the PA outcomes, adjusting for city, other socio-demographic factors (employment and marital status), and the design variables, administrative-unit-level socio-economic status and walkability. Fully-adjusted (all variables entered) GAMMs were estimated. For all main effects, a two-tailed probability level of 0.05 was adopted. Curvilinear associations of age with PA outcomes were estimated using non-parametric thin-plate splines in GAMMs [20]. Smooth terms failing to provide sufficient evidence of a curvilinear relationship (based on a 10-unit difference in Akaike Information Criterion; AIC) were replaced by simpler linear terms [20]. Separate GAMMs were run to estimate city by socio-demographic factor (age, educational attainment, and sex) interaction effects. This was done by adding two-way interaction terms to the main effect models. In the presence of curvilinear relationships (e.g., when examining the moderating effects of city on age-PA relationships), the significance of an interaction effect was evaluated by comparing AIC values of models with and without a specific interaction term(s). In such case, an interaction effect was deemed significant if it yielded an AIC 10 or more units smaller than the main effect model, indicating no support for the simpler model [22]. For city by categorical socio-demographic factor interaction effects and in the presence of a linear relationship between age and a PA variable, the significance of the specific interaction effect was determined using F-tests comparing the fit of the models with and without the interaction terms. A two-tailed significance level of 0.05 was adopted for these analyses. Significant interaction effects were probed by computing city-specific associations using linear combinations of regression coefficients based on the pooled data. Given that fewer than 5% of cases (3.72%; *n* = 512) had missing data, data analyses were performed on complete cases [22]. All analyses were conducted in R version 3.2.1 [23] using the packages ‘car’ version 2.0.26 [24], ‘mgcv’ version 1.8.7, and ‘gamm4’ version 0.2.3 [20], and ‘gmodels’ version 2.16.2 [25].

## Results

Among the 13,745 participants, the average age was 42 years old, and nearly half were males (43%) and had a college degree or higher (44%). The majority of the pooled sample reported working (74%), living with a partner (59%), and participating in any transport (76%) or leisure-time PA (71%). Study site was associated with transport and leisure-time PA. Overall, 41% of participants accumulated > 150 min/week of transport PA, ranging from 16% in Waitakere (NZ) to 65% in Aarhus. Overall, 45% of participants accumulated > 150 min/week of leisure-time PA, ranging from 29% in Cuernavaca (MX) to 72% in Aarhus (DK). All descriptive characteristics by cities are shown in Table 1.

**Table 1:** Overall and site-specific sample characteristics: socio-demographics and physical activity (PA) outcomes

	ALL SITES	AU	BE	BR	CO	CZ		DK	HK	MX	NZ				ES	UK	US	
						City A	City B				City C	City D	City E	City F			City G	City H
Overall N^1^	13,745	2650	1166	697	963	330	167	642	495	677	511	512	496	495	904	843	1287	912
Mean age (SD) missing: 1.5%	42 (12.8)	44 (12.3)	43 (12.6)	41 (13.2)	40 (13.7)	38 (14.7)	34 (13.1)	39 (13.9)	43 (11.7)	42 (12.6)	41 (11.8)	41 (11.8)	39 (12.6)	42 (12.6)	39 (14.2)	43 (13.3)	44 (11.0)	47 (10.7)
Sex, % men missing: 0.3%	43	36	48	47	36	37	40	43	41	45	36	39	49	44	45	44	55	48
Education, % missing: 1.2%																		
Less than HS	17	24	4	29	36	22	17	8	37	43	4	5	1	11	7	34	1	2
HS graduate	38	30	35	32	42	46	57	44	23	29	58	64	47	57	35	52	36	30
College or more	44	46	61	39	22	32	26	48	40	28	38	31	52	32	58	14	63	68
Work status, % working missing: 0.3%	74	71	80	78	58	77	84	75	66	72	78	84	87	80	72	64	81	83
Marital status, %couple missing: 1.2%	59	57	73	58	53	58	47	65	58	65	70	74	57	55	53	45	63	60
Any > 10 min/week transport-related PA, % missing: 0.4%	76	76	72	79	91	84	87	86	79	90	66	64	86	61	89	65	69	68
Any > 10 min/week leisure-time PA, % missing: 0.5%	71	70	74	55	55	76	78	90	68	52	71	74	84	67	86	57	80	73
Non-zero mean min/week transport-related PA^2^ (SD) n = 10,510	283 (365)	273 (358)	199 (211)	207 (304)	338 (424)	486 (499)	424 (457)	364 (407)	308 (328)	336 (485)	144 (204)	148 (251)	229 (256)	172 (227)	369 (363)	333 (423)	266 (374)	259 (315)
Non-zero mean min/week leisure-time PA^2^ n = 9734	312 (322)	294 (313)	239 (250)	245 (257)	301 (333)	428 (356)	473 (421)	462 (408)	267 (290)	277 (276)	240 (249)	221 (233)	322 (305)	249 (221)	437 (398)	348 (351)	307 (301)	295 (307)
Transport-related PA, % accumulating ≥ 150 min/week missing: 0.4%	41	39	35	30	54	65	60	59	51	45	21	16	45	22	63	35	35	35
Leisure-time PA, % accumulating ≥ 150 min/week missing: 0.5%	45	43	42	32	32	59	59	72	38	29	40	38	56	41	66	38	52	45

^1^ N for some variables is reduced due to missing data. City A: Olomouc, B: Hradec Králové, C: North Shore, D: Waitakere, E: Wellington, F: Christchurch, G: Seattle, H: Baltimore

^2^ 1% top values truncated to the maximal value of 99% of the sample (for transport-related PA: 2100 min/week; for leisure-time PA: 1680 min/week); n = number of participants with non-zero values of a specific PA

### Associations of age, educational attainment, and sex with physical activity – main effects

**Fig. 1:**
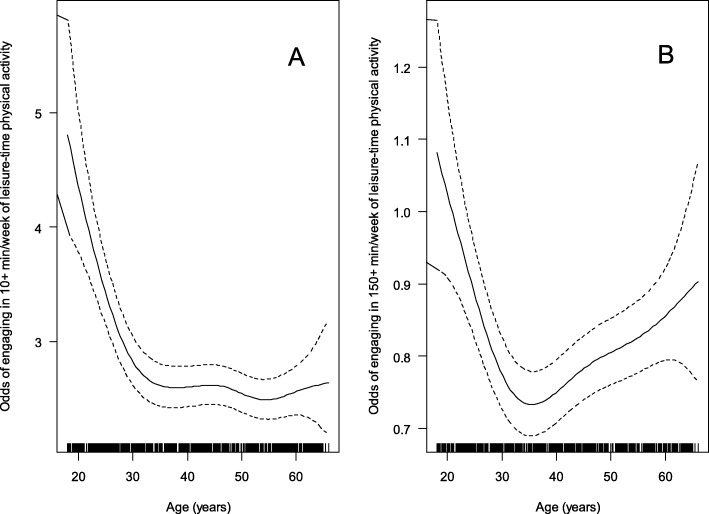
Relationships of age with the odds of engaging in ≥10 (panel A) and ≥ 150 (panel B) weekly minutes of leisure-time physical activity

**Fig. 2:**
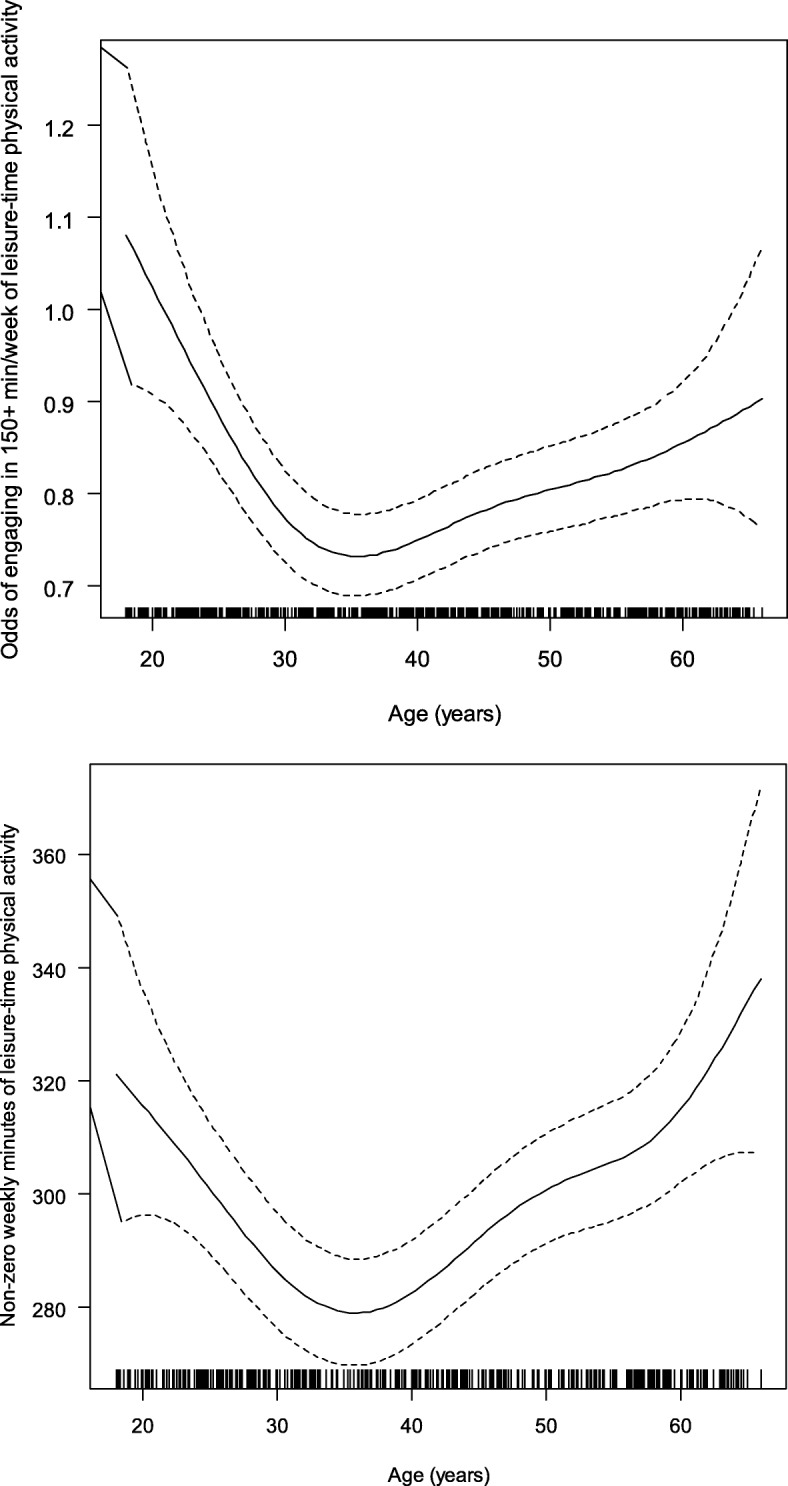
Relationship of age with the odds of engaging in ≥150 weekly minutes of leisure-time physical activity

Age was significantly but complexly associated with all PA outcomes (*p* < 0.01) (Table 2; Figs. 1 and 2), with the exception of a non-significant association for engaging in ≥150 min/week of transport PA. A linear negative association was observed with the odds of engaging in ≥10 min/week of transport PA (OR = 0.991; 95% CI: 0.987, 0.994; see Table 2), but a positive association was found with non-zero weekly minutes of transport PA in those adults (*n* = 10,166) who reported engaging in this domain of PA.

**Table 2:** Associations of age, educational attainment and sex with physical activity (PA) outcomes: Main effects for entire sample

PA outcome	n	Age	Educational attainment (reference: < high school graduate)		Sex (reference: male)
			High school graduate or some college	College degree or higher	Female
		e^b^ (e^95% CI^)	e^b^ (e^95% CI^)	e^b^ (e^95% CI^)	e^b^ (e^95% CI^)
Transport-related PA (no significant curvilinear associations)					
Yes, ≥10 min/week ^a^	13,233		F(2, 13.208) = 7.42***		
linear:		0.991 (0.987, 0.994)***	0.95 (0.83, 1.09)	1.15 (1.00, 1.33)	1.02 (0.94, 1.11)
Non-zero min/week ^b^	10,166		F(2, 10,141) = 9.24***		
linear:		1.003 (1.001, 1.004)**	1.00 (0.94, 1.06)	0.89 (0.84, 0.95)**	0.87 (0.84, 0.91)***
Yes, ≥150 min/week ^a^	13,233		F(2, 13,208) = 0.23		
linear:		1.001 (0.998, 1.004)	1.04 (0.93, 1.17)	1.02 (0.91, 1.16)	0.90 (0.84, 0.98)**
Leisure-time PA					
Yes, ≥10 min/week ^a^	13,233		F(2, 13,209) = 146.32***		
linear:		0.750 (0.549, 1.025)	1.51 (1.35, 1.70)***	2.16 (1.90, 2.45)***	0.90 (0.83, 0.97)**
curvilinear (smooth term) ^c^:		χ^2^(3.94) = 46.96*** (see Fig. 1a)	–	–	–
Non-zero min/week ^b^	9427		F(2, 9403) = 3.84*		
linear:		1.025 (0.854, 1.231)	1.02 (0.96, 1.09)	0.95 (0.89, 1.02)	0.87 (0.84, 0.91)***
curvilinear (smooth term) ^c^:		F(3.68, 9399.30) = 5.42*** (see Fig. 1b)	–	–	–
Yes, ≥150 min/week ^a^	13,233		F(2, 13,209) = 81.11***		
linear:		0.889 (0.697, 1.134)	1.43 (1.27, 1.61)***	1.75 (1.55, 1.98)***	0.87 (0.81, 0.94)***
curvilinear (smooth term) ^c^:		χ^2^(3.53) = 18.56*** (see Fig. 2)	–	–	–

95% CI = 95% confidence interval; exp.(b) exponential function of regression coefficient; exp.(95% CI) = exponential function of confidence interval; n = sample size

^a^ Generalized additive mixed model (GAMM) with binomial variance and logit link functions, for which exp.(b) is interpreted as odds ratio

^b^ GAMM with Gamma variance and logarithmic link functions, for which exp.(b) is interpreted as the proportional increase in PA associated with a 1 unit increase in the predictor

^c^ The approximate statistical significance of the smooth term is based on chi-squared (χ^2^) and F-ratio statistic for GAMMs with binomial and Gamma variance function, respectively. All models adjusted for city, working status, marital status, administrative-unit-level socio-economic status and walkability

* *p*-value ranging from ≤.05 to >.01; ** *p*-value ranging from ≤01 to >.001; *** *p* ≤ .001

In contrast to transport PA, age was non-linearly related to the odds of engaging in ≥10 min/week of leisure-time PA (Fig. 1 panels A). An inverted-U relationship was observed between age and non-zero weekly minutes of leisure-time PA in those who reported engaging in this type of activity (*n* = 9427), with the average amount of leisure-time PA decreasing from ~ 320 to ~ 280 min/week from 18 to 35 years of age and then steadily increasing (Fig. 1; panel B). The dose-response relationship between age and the odds of engaging in ≥150 min/week of leisure-time PA was similar in shape to that for the odds of engaging in ≥10 min/week of leisure-time PA (Fig. 2).

Those with a college degree were significantly more likely to engage in any transport PA than those who completed high school or had some college education (OR = 1.214; 95% CI: 1.099, 1.342; *p* < 0.001). In contrast, educational attainment was negatively related to non-zero weekly minutes of transport PA. Specifically, those with a college degree reported 11% (95% CI: 5, 16%) fewer minutes than those with less education (Table 2). Overall, educational attainment was positively related to the odds of engaging in ≥10 and ≥ 150 min/week of leisure-time PA (Table 2). However, among participants who engaged in leisure-time PA, those with high school education tended to accumulate more non-zero weekly minutes of leisure-time PA than those with a college or higher degree (e^b^ = 1.067; e^95% CI^: 1.019, 1.117; *p* = 0.005).

Sex showed a significant association with all PA outcomes with the exception of ≥10 min/week of transport PA (Table 2). For both domains, females were less likely to engage in PA and reported fewer non-zero minutes of activity compared to males.

### Moderating effects of city on associations of age, educational attainment, and sex with physical activity outcomes

City moderated associations of age with four out of six PA outcomes (Table 3). Specifically, age was significantly negatively related to the odds of engaging in ≥10 min/week of transport PA in nine cities and positively related in three cities, while no significant associations were found in the remaining five cities (Table 3). Age was positively related to non-zero weekly minutes of transport PA (in those who reported at least 10 min of this type of PA) in Ghent (BE), Olomouc (CZ), Aarhus (DK) and Pamplona (ES), and age was non-significantly related in other cities. Age was significantly negatively related to the odds of engaging in ≥150 min/week of transport PA only in Wellington (NZ) and positively related in Hong Kong and Pamplona (ES). Negative associations of age with the odds of engaging in ≥10 min/week of leisure-time PA were found in eight cities, while only Pamplona (ES) showed a weak positive association (Table 3).

**Table 3:** City-specific associations of age with physical activity (PA) outcomes

City	≥10 min/week of transport PA^a^	Non-zero min/week of transport PA^b^	≥150 min/week of transport PA^a^	≥10 min/week of leisure-time PA^a^
	F(17, 13,192) = 3.27***	F(17, 10,125) = 12.99***	F(17, 13,192) = 3.41***	F(17, 13,192) = 70.78***
	e^b^ (e^95% CI^)	e^b^ (e^95% CI^)	e^b^ (e^95% CI^)	e^b^ (e^95% CI^)
Adelaide (AU)	0.991 (0.986, 0.996)***	1.001 (0.997, 1.004)	1.000 (0.993, 1.006)	0.995 (0.987, 1.002)
Ghent (BE)	0.985 (0.980, 0.991)***	1.007 (1.002, 1.012)*	1.003 (0.993, 1.013)	0.996 (0.986, 1.007)
Curitiba (BR)	0.993 (0.987, 1.000)*	0.996 (0.990, 1.003)	1.004 (0.991, 1.016)	1.000 (0.988, 1.011)
Bogota (CO)	1.014 (1.007, 1.022)***	1.000 (0.995, 1.004)	1.003 (0.994, 1.013)	0.985 (0.975, 0.994)**
Olomouc (CZ)	0.998 (0.988, 1.007)	1.018 (1.009, 1.027)***	0.995 (0.978, 1.013)	0.971 (0.951, 0.990)***
Hradec Králové (CZ)	1.009 (0.994, 1.025)	1.007 (0.995, 1.019)	1.008 (0.983, 1.034)	0.965 (0.938, 0.994)*
Aarhus (DK)	1.001 (0.993, 1.010)	1.013 (1.007, 1.020)***	0.998 (0.985, 1.010)	0.968 (0.949, 0.989)**
Hong Kong (HK)	0.997 (0.989, 1.005)	1.004 (0.994, 1.014)	1.024 (1.006, 1.043)**	1.009 (0.989, 1.029)
Cuernavaca (MX)	1.011 (1.003, 1.019)**	0.997 (0.991, 1.003)	1.002 (0.990, 1.014)	0.979 (0.966, 0.991)***
North Shore (NZ)	0.979 (0.972, 0.987)***	0.992 (0.983, 1.001)	0.992 (0.973, 1.010)	1.000 (0.983, 1.018)
Waitakere (NZ)	0.979 (0.971, 0.986)***	1.003 (0.994, 1.011)	0.982 (0.962, 1.002)	0.991 (0.974, 1.008)
Wellington (NZ)	1.001 (0.992, 1.010)	0.997 (0.990, 1.005)	0.973 (0.958, 0.989)**	0.980 (0.961, 0.999)*
Christchurch (NZ)	0.975 (0.968, 0.982)***	0.997 (0.988, 1.006)	0.989 (0.971, 1.006)	1.006 (0.990. 1.021)
Pamplona (ES)	1.010 (1.002, 1.017)*	1.010 (1.006, 1.015)***	1.027 (1.016, 1.037)***	1.016 (1.002, 1.029)*
Stoke-on-Trent (UK)	0.983 (0.977, 0.989)***	0.997 (0.991, 1.003)	0.995 (0.984, 1.006)	0.995 (0.984, 1.006)
Seattle (US)	0.982 (0.977, 0.988)***	1.003 (0.997, 1.009)	1.001 (0.990, 1.012)	0.985 (0.972, 0.997)*
Baltimore (US)	0.984 (0.978, 0.990)***	1.002 (0.995, 1.008)	0.997 (0.984, 1.010)	0.979 (0.965, 0.993)**

95% CI = 95% confidence interval; exp.(b) exponential function of regression coefficient; exp.(95% CI) = exponential function of confidence interval

^a^ Generalized additive mixed model (GAMM) with binomial variance and logit link functions, for which exp.(b) is interpreted as odds ratio

^b^ GAMM with Gamma variance and logarithmic link functions, for which exp.(b) is interpreted as the proportional increase in PA associated with a 1 unit increase in the predictor. All models adjusted for working status, marital status, administrative-unit-level socio-economic status and walkability

* *p*-value ranging from ≤.05 to >.01; ** *p*-value ranging from ≤01 to >.001; *** *p* ≤ .001

City moderated associations of educational attainment and non-zero minutes of leisure-time PA only (Table 4). Negative associations were found in Aarhus (DK), Hong Kong, and Pamplona (ES), while positive associations were observed in Bogota (CO), Cuernavaca (MX), and Waitakere (NZ) (Table 4).

**Table 4:** City-specific associations of educational attainment with non-zero weekly minutes of leisure-time physical activity

City	Educational attainment (reference: < high school graduate)	
	F(34, 9368) = 2.35***	
	High school graduate or some college	College degree or higher
	e^b^ (e^95% CI^)	e^b^ (e^95% CI^)
Adelaide (AU)	1.09 (0.95, 1.25)	1.02 (0.90, 1.16)
Ghent (BE)	1.05 (0.74, 1.49)	0.96 (0.68, 1.35)
Curitiba (BR)	1.10 (0.84, 1.44)	1.01 (0.78, 1.31)
Bogota (CO)	0.99 (0.81, 1.22)	1.44 (1.13, 1.81)**
Olomouc (CZ)	0.80 (0.57, 1.14)	0.74 (0.51, 1.08)
Hradec Králové (CZ)	0.98 (0.56, 1.71)	1.13 (0.61, 2.06)
Aarhus (DK)	0.73 (0.52, 1.03)	0.69 (0.49, 0.98)*
Hong Kong (HK)	0.91 (0.66, 1.26)	0.73 (0.54, 0.97)*
Cuernavaca (MX)	1.21 (0.94, 1.57)	1.32 (1.02, 1.70)*
North Shore (NZ)	1.27 (0.68, 2.36)	1.10 (0.59, 2.05)
Waitakere (NZ)	1.62 (1.00, 2.61)*	1.82 (1.11, 2.98)*
Wellington (NZ)	2.44 (0.81, 7.42)	2.13 (0.70, 6.47)
Christchurch (NZ)	1.41 (0.95, 2.09)	1.52 (0.99, 2.30)
Pamplona (ES)	0.87 (0.64, 1.17)	0.64 (0.48, 0.85)**
Stoke-on-Trent (UK)	0.85 (0.69, 1.05)	0.87 (0.66, 1.15)
Seattle (US)	0.75 (0.42, 1.34)	0.66 (0.37, 1.17)
Baltimore (US)	0.88 (0.49, 1.58)	0.81 (0.45, 1.45)

95% CI = 95% confidence interval; exp.(b) exponential function of regression coefficient; exp.(95% CI) = exponential function of confidence interval. Generalized additive mixed model (GAMM) with Gamma variance and logarithmic link functions, for which exp.(b) is interpreted as the proportional increase in PA associated with a 1 unit increase in the predictor. GAMMs adjusted for working status, marital status, administrative-unit-level socio-economic status and walkability

* *p*-value ranging from ≤.05 to >.01; ** *p*-value ranging from ≤01 to >.001; *** *p* ≤ .001

City moderated associations of sex with three PA outcomes; engagement in ≥10 min/week of leisure-time PA and non-zero weekly minutes of transport and leisure-time PA (Table 5). Females were more likely than males to engage in ≥10 min/week of leisure-time PA in Ghent (BE) and Aarhus (DK), and females were less likely to do so in Adelaide (AU) and Bogota (CO). On average, compared to males, females accumulated fewer non-zero weekly minutes of leisure-time PA in four out of 17 cities and fewer non-zero weekly minutes of transport PA in five cities (Table 5).

**Table 5:** City-specific associations of sex (reference category: male) with physical activity (PA) outcomes

City	Non-zero min/week of transport PA^b^	≥10 min/week of leisure-time PA^a^	Non-zero min/week of leisure-time PA^b^
	F(17, 10,125) = 3.86***	F(17, 13,192) = 3.04***	F(17, 9384) = 4.36***
	Sex: Female	Sex: Female	Sex: Female
	e^b^ (e^95% CI^)	e^b^ (e^95% CI^)	e^b^ (e^95% CI^)
Adelaide (AU)	0.86 (0.77, 0.96)**	0.76 (0.62, 0.92)**	0.89 (0.81, 0.98)*
Ghent (BE)	0.98 (0.84, 1.16)	1.38 (1.05, 1.80)*	0.89 (0.78, 1.01)
Curitiba (BR)	0.69 (0.57, 0.84)***	1.41 (0.98, 2.03)	0.98 (0.81, 1.19)
Bogota (CO)	0.77 (0.65, 0.91)**	0.61 (0.37, 0.99)*	0.61 (0.52, 0.72)
Olomouc (CZ)	1.09 (0.79, 1.52)	1.42 (0.71, 2.86)	1.07 (0.81, 1.42)
Hradec Králové (CZ)	0.93 (0.62, 1.40)	0.83 (0.29, 2.35)	1.09 (0.76, 1.56)
Aarhus (DK)	0.99 (0.80, 1.21)	1.69 (1.06, 2.71)*	0.99 (0.84, 1.17)
Hong Kong (HK)	1.08 (0.82, 1.43)	0.81 (0.48, 1.39)	0.95 (0.74, 1.21)
Cuernavaca (MX)	0.62 (0.51, 0.75)***	1.22 (0.73, 2.02)	0.83 (0.67, 1.03)
North Shore (NZ)	0.82 (0.62, 1.07)	1.43 (0.97, 2.11)	0.88 (0.71, 1.10)
Waitakere (NS)	0.83 (0.64, 1.09)	1.15 (0.79, 1.66)	0.83 (0.68, 1.01)
Wellington (NZ)	0.97 (0.77, 1.21)	0.96 (0.57, 1.60)	0.99 (0.82, 1.19)
Christchurch (NZ)	0.76 (0.58. 1.00)	1.23 (0.85, 1.79)	0.93 (0.75, 1.16)
Pamplona (ES)	0.92 (0.78, 1.09)	1.04 (0.68, 1.60)	0.76 (0.66, 0.87)***
Stoke-on-Trent (UK)	1.08 (0.89, 1.32)	1.06 (0.80, 1.42)	0.81 (0.68, 0.97)*
Seattle (US)	0.85 (0.73, 0.99)*	0.93 (0.73, 1.19)	0.87 (0.77, 0.98)*
Baltimore (US)	1.10 (0.84, 1.22)	0.85 (0.63, 1.13)	0.99 (0.85, 1.15)

95% CI = 95% confidence interval; exp.(b) exponential function of regression coefficient; exp.(95% CI) = exponential function of confidence interval

^a^ Generalized additive mixed model (GAMM) with binomial variance and logit link functions, for which exp.(b) is interpreted as odds ratio

^b^ GAMM with Gamma variance and logarithmic link functions, for which exp.(b) is interpreted as the proportional increase in PA associated with a 1 unit increase in the predictor. All models adjusted for working status, marital status, administrative-unit-level socio-economic status and walkability

* *p*-value ranging from ≤.05 to >.01; ** *p*-value ranging from ≤01 to >.001; *** *p* ≤ .001

## Discussion

These detailed international analyses present a more complex pattern of socio-demographic associations with PA for transport and leisure purposes than is apparent in the large literature on this topic that was mainly from single countries [26–29]. Table 6 provides a simplified summary of the main effects and demographic-by-city interactions across outcomes, to assist in interpretation. In the IPEN Adult study age had the most complex associations with self-report transport and leisure-time PA. Older people in the sample were less likely to report any use of transport PA, but they reported more total minutes if they used transport PA. Perhaps this pattern reflects that older people were more likely to have cars, but those who did not have cars were more dependent on walking and bicycling. Studies of transport PA by age are rare, so the present results add new information that needs to be replicated.

**Table 6:** Summary table of results: Main effects are shown for all socio-demographic factors by physical activity variables combinations. City-specific effects are shown when there were significant demographic by city interactions

	≥10 min/week of transport PA	≥150 min/week of transport PA	Non-zero transport PA min/week	≥10 min/week of leisure PA	≥150 min/week of leisure PA	Non-zero leisure PA min/week
Age	[−]*** [−] Adelaide, Ghent, Curitiba, North Shore, Waitakere, Christchurch, Stoke-on-Trent, Seattle, Baltimore [+] Cuernavaca, Pamplona	X [−] Wellington [+] Hong Kong, Pamplona	[+]** [+] Ghent, Olomouc, Aarhus, Pamplona	X [−] Bogota, Olomouc, Hradec Králové, Aarhus, Cuernavaca, Wellington, Seattle, Baltimore [+] Pamplona	X	X
High school or some college (reference: < high school graduate)	X	X	X	[+]***	[+]***	X [+] Waitakere
College graduate (reference: < high school graduate)	X	X	[−]**	[+]***	[+]***	X [−] Aarhus, Hong Kong, Pamplona [+] Bogota, Cuernavaca, Waitakere
Female sex (reference: male)	X	[−]**	[−]*** [−] Adelaide, Curitiba, Bogota, Cuernavaca, Seattle	[−]** [−] Adelaide, Bogota [+]Ghent, Aarhus	[−]***	[−]*** [−] Adelaide, Pamplona, Stoke-on-Trent, Seattle

[−] – negative association; [+] – positive association; X – no main effect

* *p*-value ranging from ≤.05 to >.01; ** *p*-value ranging from ≤01 to >.001; *** *p* ≤ .001

Negative associations of age with leisure-time or total PA are among the most consistent correlates in studies of adults [11, 12]. Thus, it was surprising that the only simple negative linear main effect was for the odds of engaging in ≥10 min/week of transport PA. Unlike prior studies, we evaluated non-linear associations and found all three age associations with leisure-time PA measures were significantly non-linear. Present results also differed from previous global status report analyses [10]. Thus, we encourage other investigators to explore non-linear associations in their data. A possible environmental explanation is that different age groups prioritize different factors in selecting places to live, perhaps with younger adults choosing neighborhoods in suburban areas perceived as safer for children but that have fewer recreational facilities for adults. Older adults may prefer more walkable neighborhoods where they can access recreation and community centers where they could socialize with neighbors more easily [30]. Life stage may also explain the curvilinear associations with younger adults reducing leisure time activities to spend more time building their careers and raising children. As children leave home and careers are more stable, perhaps middle-aged people are able to spend more time in leisure PA.

Interactions of age and city were significant for three transport and one leisure-time PA outcomes. For ≥10 min/week of both transport and leisure-time PA, over half of cities had negative associations, and only three cities (Bogota, Cuernavaca and Pamplona) had positive associations. For non-zero minutes of transport PA, four cities had significant positive associations, with no negative associations. Though some of these divergent findings across cities could be due to modest sample sizes, it would be valuable for future studies to attempt to both confirm and explain city-specific age trends in transport and leisure-time PA related to social and built environment attributes. For instance, in Latin America a positive association between access public transit and minutes of transport physical activity have been reported among adults [31]. This association is reported in cities with low levels of car ownership and where walking to and from public transit access points is frequent.

Education had the fewest associations with PA in the present study, almost exclusively main effects of positive associations with categorical (no or yes) leisure-time PA. When comparing the college/university educated to those with less than high school, three European cities had negative associations and three cities had positive associations. Only non-zero minutes of transport activity had a negative main effect association for college educated adults, possibly because they could afford to live closer to destinations or transit. The general lack of association of education with transport PA was somewhat surprising. Although educational attainment is not an ideal proxy for income or overall SES, many studies show greater use of active travel modes by people with lower education [30, 32–35]. This pattern does not appear to generalize across countries. Transport PA may be concentrated among less-educated groups in countries with high automobile dependence, lower-quality public transport systems, and high income inequality. Perhaps income is a poorer proxy for SES in some countries where, for example, there are fewer high-paying jobs for well-educated people or where less-educated people often succeed in business. Socioeconomic inequalities are directly related to transport and leisure-time PA [26, 36], so present results might have been different if household income data had been available for inclusion. More international studies on the relation of SES to transport PA are needed.

Present results reinforce the consistent findings that education is positively related to leisure-time PA [11, 12]. These results suggest generalizability of this pattern because there was little evidence of differences by city. Only the continuous measure of non-zero minutes of leisure-time activity had a significant interaction with city, but there were few cities with significant associations, and these showed inconsistent directions. Better-educated adults are likely to have more discretionary time for leisure-time PA, more available resources such as health clubs and exercise classes, and less-active jobs that motivate them to seek leisure-time PA.

The finding that females consistently undertook less transport and leisure-time PA, with few exceptions by city, replicates one of the most common findings in the international PA literature [7, 12, 14, 37]. The generalizability of this pattern across PA domains and across geographies is of particular concern because of the implications for increased NCD risk in the female half of the world population. The sex differences were most pronounced and most consistent at the highest levels (> 150 min/week) of transport and leisure-time PA. There were some differences by city, usually reflecting a lack of significant differences by sex in some cities, but this could be partly explained by modest sample sizes. An encouraging finding was that females were more likely than males to report ≥10 min/week of leisure-time PA in Ghent (BE) and Aarhus (DK), which could reflect cultural differences that should be studied in more depth. Present findings bring new urgency to the often-stated need for improved targeting of PA interventions for females [7, 37].

## Strengths and limitations

Socio-demographic correlates are widely studied, but the present study added to the literature by providing estimates of domain-specific PA correlates across diverse countries. We examined both linear and curvilinear associations with binary and continuous leisure-time and transport PA outcomes, yielding important new information on the shape of associations of age with PA. Limitations of the study included the cross-sectional design, small number of countries with no low-income countries, samples that were not designed to be representative of populations, and the fact that sample sizes differed across countries resulting in varying power to detect city-specific associations. Although IPAQ has the strength of providing domain-specific PA estimates, it is documented that PA is over-reported with IPAQ [38]. Among other limitations are self-reported PA, analysis of only two PA domains, reporting of bouts of at least 10 min, and inability to examine total reported PA. The association between educational attainment and physical activity might have been clarified if household income data were available.

## Conclusions

The present study examined socio-demographic correlates of PA for transport and leisure-time purposes, and international variations were documented by age, education, and sex. These results can be used to inform targeting of interventions to subgroups at high risk for physical inactivity. A key finding was the generalizability of lower PA of females across leisure and transport domains and country. Tailoring PA interventions to the needs and interests of women should be a higher global public health priority. Education was primarily related positively to leisure-time PA, so interventions targeting this domain should be tailored to the needs of groups with lower education, and greater investment in interventions delivered to communities with low education are likely to be needed. Previous studies emphasized linear declines in PA with age, but present findings documented consistent curvilinear associations with leisure-time PA. This novel finding should be further investigated to determine how consistent it is using such non-linear analyses.

## Data Availability

As the consent forms indicated that the data would be only accessible to the team of investigators, the data are confidential. De-identified data are however available from the authors upon reasonable request and with permission of national study coordinators.
